# Dr. William Tillinghast Bull

**Published:** 1909-03

**Authors:** 


### OBITUARY

Dr. William Tillinghast Bull, of New York, died at Wymberly, Isle of Hope, Ga., at noon February 22, 1909, aged 60 years. He had been ill for several months with a hopeless disease—cancer. He went south a few weeks ago to get the benefit of a milder climate, and thus stay in the open air for a considerable portion of each day. According to official announcement the end was due to edema of the lungs. For the last few days his condition gradually had grown weaker.

William Tillinghast Bull was born in Newport, R. I., in 1849. His first American ancestor was one of nine original settlers at Aquidneck, which later became Newport. He was educated at Harvard, the medical department of Columbia University, and in the hospitals of Europe. From the time he began to practise, in 1877, until illness incapacitated him, he was connected with various New York hospitals.

Dr. Bull became famous while in charge of the Chambers Street Hospital. He went to this institution in 1877 and remained there until 1888. His remarkable work in emergency cases gave this hospital a wide reputation. It was while here that, by an improved method of laparotomy, he helped to revolutionise the treatment of gunshot wounds in the abdomen. His method of treatment greatly reduced the mortality, which had been about 87 per cent. In 1889, after a competitive examination, he was appointed professor of surgery at the College of Physicians and Surgeons of Columbia University—a position he held until illness overcame him. At the time of his death and for some years previously, Dr. Bull easily took rank among the foremost surgeons of the world.

